# A blended e-health intervention for improving functional capacity in elderly patients on haemodialysis: A feasibility study

**DOI:** 10.3389/fdgth.2022.1054932

**Published:** 2022-12-06

**Authors:** Damiano D. Zemp, Pierrette Baschung Pfister, Ruud H. Knols, Pierluigi Quadri, Giorgia Bianchi, Davide Giunzioni, Soraya Lavorato, Olivier Giannini, Eling D. de Bruin

**Affiliations:** ^1^ Geriatric Service, Ospedale Regionale di Mendrisio, EOC, Mendrisio, Switzerland; ^2^ Institute of Human Movement Sciences and Sport, Department of Health Sciences and Technology, ETH Zurich, Zurich, Switzerland; ^3^ Directorate of Research and Education, Physiotherapy Occupational Therapy Research Center, University Hospital Zurich, Zurich, Switzerland; ^4^ Department of Physiotherapy and Occupational Therapy, University Hospital Zurich, Zurich Switzerland; ^5^ Department of Internal Medicine, Ente Ospedaliero Cantonale, Bellinzona, Switzerland; ^6^ Division of Nephrology, Ente Ospedaliero Cantonale, Lugano, Switzerland; ^7^ Faculty of Biomedical Sciences, Università Della Svizzera Italiana, Lugano, Switzerland; ^8^ Department of Neurobiology, Care Sciences and Society, Karolinska Institute, Stockholm, Sweden; ^9^ OST – Eastern Swiss University of Applied Sciences, Department of Health, St. Gallen, Switzerland

**Keywords:** tablet-computer, exercise, haemodialysis, elderly, e-health, blended intervention, physiotherapy

## Abstract

**Introduction:**

Physical exercise showed to be beneficial for frail older adults on haemodialysis (HD). However, there are several obstacles hindering the regular practice of exercise, such as transportation difficulties, lack of time, fatigue and comorbidities. E-health in this regard has many potential advantages and could be useful for motivating HD patients to increase their level of physical activity. The aim of this study was to evaluate the feasibility of a blended e-health intervention for elderly HD patients who individually exercise at home while under remote supervision of a physiotherapist.

**Material and methods:**

Patients over 60 years of age with sufficient cognitive and motoric resources to perform a simple physical test battery and to use a tablet-computer were recruited from four HD outpatient facilities. Following baseline assessment at home, the participants were visited by a physiotherapist (PT). The PT set an individual exercise programme and explained how to use the web-based interface. During the 12 weeks of training, the PTs remotely supervised the patients' progress. At 12 weeks follow-up a second assessment took place.

**Results:**

Twenty-two patients were recruited to participate in the study. Seven patients dropped out of the blended programme and 15 patients concluded the programme. The average training frequency of the 15 participants concluding the study was 1.5 times a week [range 0.2–5.8]. The duration of a training session was between 20 and 40 min. The usability of the system was deemed positive. Regarding the efficacy of the intervention, no significant improvement of any measured parameter was found, and effect sizes were small to medium.

**Conclusion:**

A blended e-health intervention supported by a web-based application for exercising at home under remote supervision of a PT is feasible in a HD population including older patients. However, before planning a randomized controlled trial, strategies to increase the recruitment rate and the adherence to such a blended intervention should be further developed, e.g., to improve the recruitment procedures and lower the expectable drop-out rate. Furthermore, the dosage of the blended programme should be adapted to the patients' physical performance levels in future trials.

The study was registered on the website clinicaltrials.gov with ID NCT04076488.

## Introduction

Haemodialysis (HD) is the most frequent renal replacement therapy used in patients with end stage kidney disease (1, 2). HD is a time-consuming medical treatment that requires mostly three sessions weekly for up to four hours in an ambulatory hospital setting or a clinic dialysis facility. Due to the chronic nature of the disease and the older mean age of the affected persons (1, 3, 4), the HD population shows a high prevalence of falls (5, 6) and of frailty (7).

Despite the reported health benefits of physical exercise programmes in HD patients (8–11), the adherence to such programs is known to be hindered – especially in the more older patients – by a number of barriers such as low energy and fatigue, lack of time, medical problems or transportation difficulties (12–16).

Since the number of people on HD is small (about 4,700 in Switzerland (17)), no specific training for physiotherapists is available in Switzerland, and the patient specific expertise in this field is limited to the reference HD centres. Furthermore, to the best of our knowledge, the available guidelines in this field are only taking “adults” into account, and are not always entirely applicable to elderly patients (18). Disease specific exercise guidelines are missing (19).

Access to high quality care is often limited in elderly patients because of travel distance and related travel costs (20) that in Switzerland are not covered by the health insurance. Overcoming these barriers and beginning an exercise training programme is, however, considered the basis for a successful intervention in which currently available evidence-based best practice is applied (10). When patients start participation in an exercise programme, a further challenge is the maintenance of high adherence to the training over longer time periods (21).

E-health refers to health services delivered through the internet or related technologies (22). A blended e-health intervention (a combination of face-to-face care with online care (23)) may help to overcome some of the barriers to regular physical exercise (24, 25). This approach may optimize the timing, the intensity and the sequencing of interventions, and provides opportunities for individuals to receive specialised care rehabilitation in their own social environments, thus enhancing the availability and capacity of rehabilitation programmes (26). Recently the potential of e-health was used for fall prevention programmes in the elderly, reporting encouraging results (27–32). An additional advantage of e-health is the possibility of implementing different persuasive technologies such as personalisation, self-monitoring, tailoring, goal setting, comparison, conditioning through positive reinforcement and remote support in the development of exercise programmes, that can enhance patients' motivation to exercise regularly (23, 33–35). When web-based and non-web-based interventions are compared this shows more improvement in the ability of individuals using web-based interventions to achieve the desired specified knowledge and/or behavioural changes (36). A web-based solution allows remote supervision and training programme modification by the treating PT. Combining the web-based intervention with face-to-face sessions has shown to improve adherence in the elderly (37).

E-health is relatively new and is considered a promising method in the treatment of patients with chronic kidney disease that should be further developed (38). The aim of this study is to evaluate the feasibility of an interactive individualized web-based blended e-health exercise programme in elderly HD patients. Furthermore, this study evaluates effects of the blended intervention on health-related parameters and functional capacities as secondary outcome.

## Material and methods

### Study design and participants

HD patients were enrolled into a quasi-experimental single group repeated measures design study over a period of 12 weeks. CONSORT guidelines for feasibility trials (39, 40) and TIDierR guidelines for intervention studies (41) are followed in this manuscript with checklists available in Supplementary Tables S1 and S2.

This is a single-arm feasibility study in which every patient that met inclusion criteria and accepted to participate in the study was prescribed a training program of 12 weeks duration.

Eligible HD patients were over 60 years old, able to walk 20 meters at a minimum without walking aids, were on a stable medical regime (e.g., no ongoing oncological treatment, no recent surgery and no acute severe disease (e.g., a not healed trauma or fracture, ongoing infections) hindering participation in a regular training programme) and did not perform more than one weekly session of vigorous physical activity (including physiotherapy sessions). The exclusion criteria were contraindications to physical exercise, known or suspected non-compliance (e.g., patients not compliant to other therapies like physiotherapy or medicine intake), drug or alcohol abuse, and cognitive impairment that led to the inability to follow the procedure of the study.

### Recruitment

We expected to enrol 3–5 patients per HD centre for a total of 12–20 patients. This is in line with a general rule-of-thumb (42) that sets the minimum of participants needed for feasibility studies at 12 (42, 43).

Initially all HD patients were screened by the medical doctors (MD) responsible for the four public HD facilities of the multicentre Hospital “Ente Ospedaliero Cantonale” of Canton Ticino, Switzerland (with centres located in the cities of Mendrisio, Lugano, Bellinzona and Locarno) during four weeks in September 2019. *P*atients who met inclusion criteria and gave their oral consent to the MD to participate were then contacted by the main investigator for organising a home visit that included the signing of the informed consent to the study and the baseline assessment in the following months.

### Intervention

The intervention was a blended therapy approach that combined face-to-face training sessions performed by a PT with an interactive web-based home exercise programme. For this purpose, the application “Fit” was used (Dividat AG, Schindellegi, Switzerland). The application works interactively, meaning that a treating PT supervises the training progress of a patient on a weekly basis *via* remote communication. The collected data about the frequency of the training session or regarding a specific exercise also allows determining whether an extra home visit is required to motivate the patient or to modify the training programme.

During the initial face-to-face session, the PT examined the patient, set up an individually tailored physical exercise programme with four to six exercises, and practised these with the patient. Before the patient embarked on the 12-week home programme, he/she received a tablet-computer that included the programmed physical exercises, and instructions on how to use the “Fit” application.

The Fit cloud-based application from Dividat AG is described in detail elsewhere (44).

Due to the fact that guidelines about exercise training in HD patients - while planning for this study - was limited to adults (18), we based the development of the exercises mainly on national and international fall prevention guidelines for elderly (45–47). Based on the needs of the patient, an individually tailored set of exercises could be provided. A pool of 34 dynamic, single- and multiple-joint exercises with different difficulty levels was available for training strength, balance, mobility, gait, and coordination. The exercise collection with the description in Italian can be made available by the authors upon reasonable request.

A minimum training frequency was set to 2 sessions per week and for a duration between 20 and 40 min. The intervention was planned to last 12 weeks. This corresponds to the minimum dose for an efficient exercise programme for fall prevention (48). An adherence of ≥ 75% was deemed acceptable and is reflective of reported adherence in people on HD who trained at home (49–52). This 75% threshold is somewhat higher compared to the 67% average adherence value for unsupervised home-based resistance training in older adults (53).

The PTs were trained in using the programme and had 2 years of experience using the system. Furthermore, the PTs were actively working in geriatric rehabilitation. The investigator who performed the baseline and the follow-up assessments was a movement scientist trained in the use of the assessments. This person had more than 10 years of working expertise in movement analysis in the geriatric population.

### Outcomes

#### Primary feasibility outcomes

The following parameters were used to analyse the feasibility of the intervention:

– Inclusion rate: the ratio of patients fulfilling the inclusion criteria to all elderly HD patients in the 4 facilities.
– Recruitment rate: the ratio of patients registered in an HD centre in September 2019 who accepted to participate to all elderly HD patients who met the inclusion criteria.
– Attrition: the ratio of participants who did not terminate the 12-weeks programme to all patients who started the exercise program.
– Adherence: the ratio of number of training sessions (registered on the cloud) to the number of possible training sessions (2 times a week for 12 weeks = 24).

Additionally, the reasons for drop-out and for low adherence were recorded.

Acceptance of “Fit” was analysed using an adapted version of the TAM (Technology Acceptance Model) questionnaire which contains 20 statements to be rated on a 7-point Likert scale ranging from 1 (strongly disagree) to 7 (strongly agree) (54–56), with a high score meaning a positive evaluation of the system. The TAM analyses the perceived ease of use (PEU), the perceived usefulness (PU), the attitude towards using (ATU), and the behavioural intention to use (BIU).

#### Secondary outcomes

Functional capacity and health related aspects were tested by a “blinded” assessor at baseline and after 12 weeks at the participant's home. HD-associated symptoms (e.g., fatigue, dizziness) are common and about 25% patients recover the next day (57). To avoid these symptoms influencing the outcome of the functional tests we set the assessment session 24 h after a HD session. The test-battery included the 4 m walk test (4mWT) without using a walking aid (58), (which was modified through the elimination of the 1 m acceleration phase because of the difficulty in finding a 6 m corridor at every participant's home), handgrip strength (using a Jamar® hydraulic hand dynamometer from Performance Health International LTD, Sutton-in-Ashfield, UK) (59, 60), the Short Physical Performance Battery (SPPB) (61, 62), the 60 s chair stand (63, 64) and the Timed Get up and Go Test (TUG) (65, 66). The latter test was taken under single-, dual-cognitive- (counting backward from 100 by steps of 3) and dual-motor-task (carrying a cup full of water) (67) conditions. The dual-task cost for the TUG (CT-cost for the cognitive and MT-cost for the motoric-dual-task) was calculated in percentage using the formula:

(100×dual−task_value:single−task_value)–100

Physical and mental health status was assessed with the Short Form Health Survey (SF-12) (68–71) and autonomy with the de Morton Mobility Index (DEMMI) (72). The assessments of the secondary outcomes are described in detail in Supplementary File S3.

### Statistical methods

We described the general characteristics, the functional capacities, the health-related characteristics of the patients, and the five aspects of the TAM questionnaire, using mean and standard deviation and reporting minimum and maximum values.

The primary feasibility outcomes were reported with narrative and descriptive statistics with absolute and relative numbers.

Although this is a feasibility study, we assessed the functional capacities before and after the intervention with the intention to describe the population and provide future researchers with data for comparison. Our goal was not to test efficacy of the intervention but, in line with guidelines for feasibility trials (73, 74), confidence intervals are provided to reflect the uncertainty of the main feasibility outcome. We compared the baseline and follow-up data in those patients who concluded the training programme, using the paired-samples t-test and reported mean difference ± standard error, 95% Confidence Interval, and effect size (calculated with Cohen's d for within-group comparisons, where 0.2 stands for a small effect, 0.5 for a medium effect, and 0.8 for a large effect size) (75). For not normally distributed data (normality tested with the Kolmogorov-Smirnov test), the bootstrapped confidence interval was reported.

Missing data, for example due to technical problems or health status of the participants, was not substituted, and no adjustment of mean and standard deviation was carried out. For statistical analysis IBM SPSS Statistics 27 was used.

## Results

Recruitment took place in September 2019. The first baseline assessments of the first patients were made on the 17th of October 2019 and the last follow-up visit on the 20th of October 2020.

Patient characteristics are presented in Table 1.

**Table 1:** Baseline demographic, clinical and functional data: mean ± SD [range] characteristics of the participants. Where not specified, all 21 participants are included.

	Participants (*n* = 21)	Reference Values
General characteristics		
Age (years)	77.2 ± 7.1 [64–90]	
Gender (M/F)	13/8	
BMI (kg/m^2^)	28.8 ± 5.8 [17.7–42.9]	18.5–24.9 kg/m^2^
Time on HD (months)	59.1 ± 57.7 [4–225]	
Functional capacity		
SPPB (points)	7.8 ± 3.2 [0–12]	> 8 points
4 m walk test (s)	5.8 ± 1 [3.9–7.9] (*n* = 19)	≤ 5 s
Handgrip (kg)		
– Female – Male	18.6 ± 3.1 [14–22]	≥ 16 kg
	30.0 ± 10.3 [8–44]	≥ 27 kg
TUG (s)	15.2 ± 5.2 [9.7–27.5]	< 14 s
60 s Chair Stand (number)	17.1 ± 7.5 [0–29] (*n* = 20)	> 22
Cognitive dual-task Cost (%)	30.2 ± 25.3 [−0.4−117]	< 20%
Motor dual-task Cost (%)	8.4 ± 11.6 [−11−40] (*n* = 19)	< 10%
Health status		
Comorbidity Severity Index^1^	0.8 ± 0.2 [0.5–1.6]	≤ 2 points
Comorbidity Index^1^	1.1 ± 0.4 [1–2]	≤ 2 points
DEMMI (points)	73.7 ± 16.5 [39–100]	> 60 points
Physical health (points)	36.8 ± 7.9 [22.1–54.2]	> 40 points
Mental health (points)	52.3 ± 9.6 [23.3–65.6]	> 40 points

BMI, Body Mass Index; SPPB, Short Physical Performance Battery; TUG, Timed Get up and Go Test; DEMMI, de Morton Mobility Index. ^1^Assessed through the Cumulative Illness Rating Scale (76–78).

### Primary feasibility outcomes

**Figure 1:**
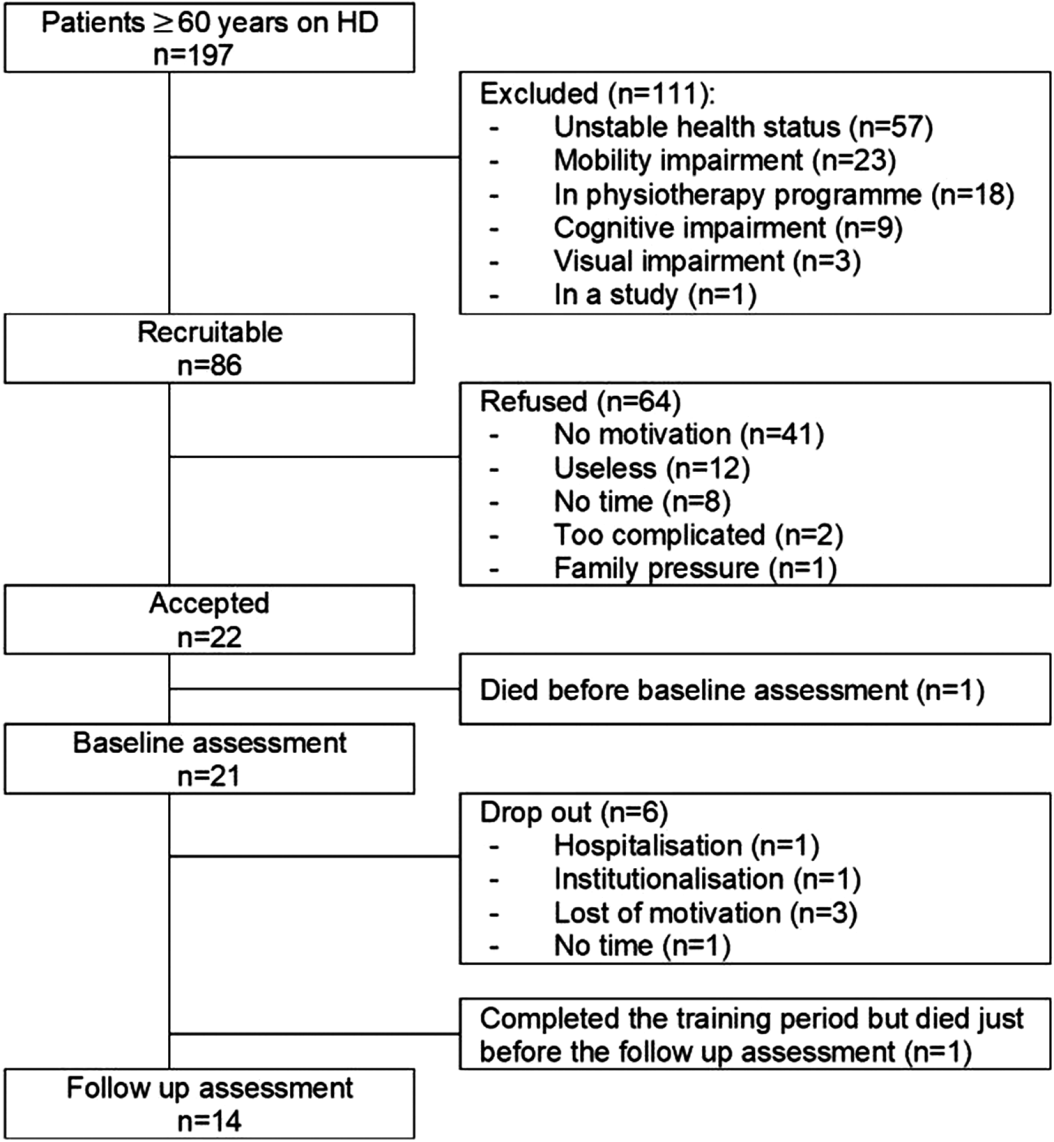
Study flow diagram.

One hundred and ninety-seven (197) HD patients over 60 years of age were screened for participation in the four centres. One hundred and eleven (111) out of the 197 patients did not fulfil inclusion criteria (56%). Out of the 86 remaining potential participants, 22 agreed to participate (26%). The main reason for not participating was lack of motivation (refusal without giving a specific explanation) to participate in an exercise programme (64%). One participant willing to participate passed away before the baseline assessment, and out of the 21 participants starting the exercise program, six dropped out (29%). Three participants abandoned the programme because of motivation loss, one was hospitalised, one was institutionalised in a care home, and one was not able to continue due to lack of time. One participant finished the training programme but died before the follow-up assessment, and another refused the follow-up visit at home due to the COVID-19 pandemic; however, this individual completed the questionnaires by phone. Figure 1 shows the study flow diagram.

The 15 participants who concluded the exercise programme had a mean adherence of 73% (ranging between 10% and 290%), whereas six had an adherence ≥ 75%. The reason for the low adherence were health-related in three cases, in two cases because of difficulties using the tablet, one participant preferred outdoor activities to the tablet solution, one participant changed jobs and had no time for training, one lost the motivation to exercise, and one had to take part in informal care. Furthermore, two patients had to undergo surgery, due to a knee injury and femur fracture (not related to the exercise program). After recovery, both patients continued with the programme with mainly exercises performed in a sitting position. The feasibility parameters are summarized in Table 2.

**Table 2:** Feasibility parameters inclusion- and recruitment rate, attrition and adherence.

Parameter	Ratio	Percentage
Inclusion rate	86/197	44%
Recruitment rate	22/86	26%
Attrition	6/21	29%
Adherence	17/24	73%

Table 3 Summary of the acceptance of the “Fit” system based on the TAM questionnaire scores.

**Table 3:** Acceptance of “Fit” as analysed with the technology acceptance model questionnaire (values represent mean ± SD of the given scores).

Category				Score	Interpretation
Perceived ease of use (PEU)				6.2 ± 0.3 [4.8–7.0]	Very high
Perceived usefulness (PU)				5.5 ± 0.4 [3.5–7.0]	Very high
Attitude towards using (ATU)				5.6 ± 0.4 [2.5–7.0]	Very high
Behavioural intention to use (BIU)				3.9 ± 0.6 [1.0–7.0]	High
Score Explanation Scale (56)					
< 1.5 = very low	1.5-2.5 = quite low	2.5-3.5 = low	3.5-4.5 = high	4.5-5.5 = quite high	≥ 5.5 = very high

### Secondary outcomes

For the functional capacity and the health status, there were no significant changes after the 12-week exercise programme. The 4 m walk test, handgrip, and the dual-task cost (both cognitive and motor) showed a small to medium effect size, the other parameters measured had a small effect size. The results are summarized in Table 4. The individual participant results are presented graphically in Supplementary Table S4.

**Table 4:** Pre and post measurements (mean ± SD) and statistics.

	*n*	Pre	Post	Mean difference ± SE [95% CI]	Effect size
Functional capacity					
SPPB (points)	13	8.7 ± 2.7	8.6 ± 3.0	−0.1 ± 0.6 [−1.3−1.1]	0.04
4 m walk (s)	12	5.6 ± 0.8	5.8 ± 1.3	0.3 ± 0.3 [−0.3−0.8]	0.27
TUG (s)	12	13.0 ± 2.3	13.1 ± 2.9	0.1 ± 0.7 [−1.4−1.7]	0.06
Handgrip (kg)					
– Female – Male	7	19.8 ± 3.0	18.8 ± 3.1	−1.0 ± 1.5 [−5.3−3.3]	0.31
	5	32.7 ± 9.1	34.6 ± 6.3	1.9 ± 1.9 [−2.8−6.5]	0.37
60 s chair rise (s)	12	20.8 ± 4.8	20.5 ± 4.8	−0.3 ± 1.2 [−2.9−2.4]	0.06
Cognitive dual-task Cost (%)*	12	30.1 ± 16.9	33.5 ± 28.1	3.5 ± 7.4 [−10.5−18.0]	0.20
Motor dual-task Cost (%)	12	6.2 ± 8.9	9.1 ± 9.9	2.9 ± 3.8 [−5.5−11.3]	0.22
Health status					
DEMMI (points)	14	80.7 ± 13.5	80.3 ± 16.0	−0.4 ± 3.3 [−7.5−6.7]	0.04
Physical health (points)	14	38.1 ± 6.6	37.6 ± 11.2	−0.6 ± 2.2 [−5.3−4.2]	0.07
Mental health (points)	14	53.9 ± 8.3	54.9 ± 7.5	1.0 ± 1.7 [−2.7−4.6]	0.16

SPPB, Short Physical Performance Battery; TUG, Timed Get up and Go Test; DEMMI, de Morton Mobility Index; SE, Standard Error; CI, Confidence Interval.

* not normally distributed.

## Discussion

The main aim of this study was to analyse the feasibility of a blended e-health intervention in elderly HD patients, and to quantify the impact of the 12-week training programme with the individualised web-based exercise programme on the functional capacity and the health status of the participants. After excluding 60% of the HD patients, mostly due to their impaired health status, 26% of the eligible patients accepted to participate in the study. 68% concluded the 12-week exercise programme with an adherence of 73%. Our results are similar to other studies that tested home-based training in HD patients (49–52, 79, 80). The application “Fit” was evaluated as useful, motivating and easy to use. No significant changes were found in functional capacity and health related aspects in the group that concluded the study.

Due to the more advanced age of the participants, the chronic nature of end stage renal disease with its specific accompanying symptoms (fatigue, comorbidities), and with the time and energy required for haemodialytic therapy, we were aware that it would be difficult to motivate HD patients to invest time and energy in an exercise programme (12–16), notwithstanding the known benefits of exercise for these patients (8, 9).

In order to motivate HD participants to exercise we combined the advantage of a home-based training approach (temporal and spatial independence) with technological motivational aspects (self- and external remote monitoring) under the supervision of specialized physiotherapists that included at least one face-to-face visit (24–26).

The initial screening process confirmed the frailty prevalence in the HD population, where out of 197 elderly patients on dialysis, 57 had an unstable health status (29%), and 35 were physically, cognitively, or visually impaired (18%). These patients could probably also benefit from the system, but for this study they were not eligible due to our protocol. This included widely used geriatric assessments that contain many walking tasks and expected the participants to use the system autonomously at home, although our experience suggests that the assistance by a family member or a care giver is in some cases useful.

Another aspect that emerged was the lack of motivation in our patient group. About 50% (41 out of 86) stated unwillingness as the reason for their refusal to participate, and 14% (12 out of 86) considered the intervention to be useless from the outset. In this regard, the role of healthcare staff seems to be fundamental, as a positive attitude of the health care staff towards physical exercise has previously shown to improve the motivation of training in their patients (81, 82). For further studies in this field, instruction of the medical staff may be useful for improving the recruitment rate (15). Only two patients motivated their refusal with an aversion towards technology. This confirms the high acceptance towards the web-based technology reported in a recent survey in Switzerland (83).

The mean adherence of the participants who concluded the 12-week programme was 73%, and is in line with similar studies that had an adherence in home-based trainings programme for HD patients of 53%–78% (49–52, 79).

Interestingly, the low adherence was not conditioned by health status alone. In fact, two participants continued to exercise regularly even after knee surgery and a hip fracture. In these cases, the training plan was adapted and sitting exercises substituted for standing exercises.

The functional capacity measured in the participants at baseline, mostly in a sub-normal range, the low physical health score, and the number of adverse events (2 deaths, 1 hospitalisation, 1 institutionalisation and 2 surgeries), confirm the frailty status of the HD population, and should be considered in further studies when estimating the drop-out rate in a similar population. An encouraging finding, however, was that no adverse event was related to the intervention.

The application “Fit” was evaluated very positively and was not an obstacle for participation in the programme. The possible technological issues that we anticipated were reduced to a minimum through providing the patients with a complete solution in which as few steps as possible were needed to set up and initiate the system. They received a tablet with the pre-installed application and a subscriber identity module (SIM card) that ensured the internet connection. In addition, the home visit provided the PT, together with the user, with the opportunity to determine how best to set up the system and where to plug the tablet in. In fact, contrary to the findings of a similar project (84), where the patients received the tablet in the hospital and had to connect the system to their own internet service at home without receiving any assistance, no technical problems were reported in our study. In line with, and as described in other publications (24, 25), the blended approach was by and large appreciated, and the supervision by the PT who contacted the participants by phone once an issue came up was positively interpreted by the trainees as a sign of close involvement.

Despite the very high score in perceived usability of the system and the perceived usefulness, only 40% of our study participants trained regularly (6 out of 15 patients). The low adherence and the adverse events (two patients underwent surgery) may be indicative of the vulnerable patient status which possibly also explains the lack of improvement in functional capacity and health status. However, it should be noted that the focus of this study was on feasibility and not on effectiveness. This is why we integrated a rather generic exercise programme instead of developing a training plan with clear SMART goals (85) targeted to the deficits of this population. Nevertheless, lifestyle changes such as the use of an e-health application may be very challenging for HD patients, and the use of education techniques, goal setting, feedback, monitoring and social support are required to prevent progression of the disease (86). Future studies that use this or similar approaches that target effectiveness should pay careful attention to the design of the training content.

The expected cost for the intervention could be an important barrier especially for people with a low income. Therefore, an analysis of the costs of such an intervention should be a topic for future trials. We estimate the costs for a tablet PC at EUR 100 and the monthly abonnement to the internet being about 10 Euros per month. The first visit of the PTs lasted about two hours whereas the remote supervision took about 15 min per week. With a salary of EUR 35 per hour the cost for the whole intervention (material and salary) we estimated a maximal cost of 350 euro for a 3 month intervention in Switzerland. Without the reimbursement at least of a part of the intervention by an health insurance it is unlikely this system can be successful. However, having said this, from a recent review it can be derived that the costs for a blended physiotherapy approach may also show to be substantially lower compared with traditional care (87).

This study can be seen as one of the first steps in developing an innovative complex intervention. The next steps needed for further development should preferably be guided by key principles of intervention development recommended by the United Kingdom medical research council (MRC) (88). The next phase would involve refining and optimising our earlier version of the blended exercise intervention. For this purpose we expect that a series of iterations should allow us to assess how acceptable, feasible and engaging the intervention is (88).

### Limitations

No analysis was carried out on the patients who refused to participate. This could have been helpful for developing further motivational strategies and improving the recruitment rate. “Fit” and the design of the study were explained to the MD who recruited the patients, but no motivational strategies were discussed. This could also have led to an improvement in the recruitment rate.

We planned only one face-to-face visit, but the PT could organise additional visits in case deemed necessary and contact the participants regularly by phone. Whether more face-to-face sessions would increase adherence is controversial. Liu-Ambrose et al. reported a lower adherence with 4 home sessions (89), whereas Kamide et al. reported a higher adherence with only one face-to-face session (90), both in a 6-month home-based training programme with older participants (not on HD).

The low number of participants and the fact that only 6 out of 15 concluders trained regularly (at least 1.5 training sessions per week) could explain the non-significant impact of the intervention on the assessed parameters. However, the focus of our trial during this stage of development was rather on feasibility and not on effectiveness. In this context a further perceived limitation of our study could be the fact that some clinical tests were adapted or were assessed using different approaches. In future trials that focus on effectiveness it is important using tests and test protocols with known psychometric properties in sufficiently large samples.

## Conclusion

If further adapted to the older HD population, a home-based exercise programme supported by a tablet and remotely supervised by a health professional may be feasible and beneficial for users who regularly exercise and are willing to be remotely monitored. Strategies to increase the perception of the benefits of physical activity and to improve the adherence to an exercise programme should be developed, including involvement of medical, nursing, and therapeutic staff. Patients could have, prior to engaging in such an unfamiliar remotely supervised blended exercise programme at home, the more complex intervention system presented to them in an HD centre.

## Data Availability

The original contributions presented in the study are included in the article/**Supplementary Material**, further inquiries can be directed to the corresponding author/s.

## References

[1] Kramer A Pippias M Noordzij M Stel VS Andrusev AM Aparicio-Madre MI The European renal association - European dialysis and transplant association (ERA-EDTA) registry annual report 2016: a summary. Clin Kidney J. (2019) 12(5):702–20. 10.1093/ckj/sfz011 31583095 PMC6768305

[2] Liyanage T Ninomiya T Jha V Neal B Patrice HM Okpechi I Worldwide access to treatment for end-stage kidney disease: a systematic review. Lancet. (2015) 385(9981):1975–82. 10.1016/S0140-6736(14)61601-9 25777665

[3] US Renal Data System 2019 Annual Data Report. Epidemiology of kidney disease in the United States. Am J Kidney Dis. (2019) 75(1-S1):S1–S64. 10.1053/j.ajkd.2019.09.002 31704083

[4] Ambuehl P. Das schweizer dialyseregister - aktuelle erkenntnisse zur schweizer dialysepopulation. Medizion Aktuell. (2017) 12(3):22–6.

[5] Abdel-Rahman EM Turgut F Turkmen K Balogun RA. Falls in elderly hemodialysis patients. Qjm. (2011) 104(10):829–38. 10.1093/qjmed/hcr108 21750022

[6] Cook WL Tomlinson G Donaldson M Markowitz SN Naglie G Sobolev B Falls and fall-related injuries in older dialysis patients. Clin J Am Soc Nephrol. (2006) 1(6):1197–204. 10.2215/CJN.01650506 17699348

[7] McAdams-DeMarco MA Law A Salter ML Boyarsky B Gimenez L Jaar BG Frailty as a novel predictor of mortality and hospitalization in individuals of all ages undergoing hemodialysis. J Am Geriatr Soc. (2013) 61(6):896–901. 10.1111/jgs.12266 23711111 PMC3938084

[8] Bronas UG Puzantian H Hannan M. Cognitive impairment in chronic kidney disease: vascular milieu and the potential therapeutic role of exercise. Biomed Res Int. (2017) 2017:2726369. 10.1155/2017/2726369 28503567 PMC5414492

[9] Müller-Ortiz H Pedreros-Rosales C Vera-Calzaretta A González-Burboa A Zúñiga-San Martín C Oliveros-Romero MS. [Exercise training in advanced chronic kidney disease]. Rev Med Chil. (2019) 147(11):1443–8. 10.4067/S0034-98872019001101443 32186605

[10] Yamagata K Hoshino J Sugiyama H Hanafusa N Shibagaki Y Komatsu Y Clinical practice guideline for renal rehabilitation: systematic reviews and recommendations of exercise therapies in patients with kidney diseases. Renal Replace Ther. (2019) 5(1):28. 10.1186/s41100-019-0209-8

[11] Mallamaci F Pisano A Tripepi G. Physical activity in chronic kidney disease and the EXerCise Introduction to enhance trial. Nephrol Dial Transplant. (2020) 35(Suppl 2):ii18–22. 10.1093/ndt/gfaa012 32162664 PMC7066543

[12] Hannan M Bronas UG. Barriers to exercise for patients with renal disease: an integrative review. J Nephrol. (2017) 30(6):729–41. 10.1007/s40620-017-0420-z 28689231 PMC8171436

[13] Jhamb M McNulty ML Ingalsbe G Childers JW Schell J Conroy MB Knowledge, barriers and facilitators of exercise in dialysis patients: a qualitative study of patients, staff and nephrologists. BMC Nephrol. (2016) 17(1):192. 10.1186/s12882-016-0399-z 27881101 PMC5121941

[14] Delgado C Johansen KL. Barriers to exercise participation among dialysis patients. Nephrol Dial Transplant. (2012) 27(3):1152–7. 10.1093/ndt/gfr404 21795245 PMC3289894

[15] Michou V Kouidi E Liakopoulos V Dounousi E Deligiannis A. Attitudes of hemodialysis patients, medical and nursing staff towards patients’ physical activity. Int Urol Nephrol. (2019) 51(7):1249–60. 10.1007/s11255-019-02179-1 31161521

[16] Kontos PC Miller KL Brooks D Jassal SV Spanjevic L Devins GM Factors influencing exercise participation by older adults requiring chronic hemodialysis: a qualitative study. Int Urol Nephrol. (2007) 39(4):1303–11. 10.1007/s11255-007-9265-z 17902035

[17] Guidotti R Pruijm M Ambühl PM. COVID-19 Pandemic in dialysis patients: the Swiss experience. Front Public Health. (2022) 10:795701. 10.3389/fpubh.2022.795701 35655466 PMC9152253

[18] Smart NA Williams AD Levinger I Selig S Howden E Coombes JS Exercise & sports science Australia (ESSA) position statement on exercise and chronic kidney disease. J Sci Med Sport. (2013) 16(5):406–11. 10.1016/j.jsams.2013.01.005 23434075

[19] Lambert K Lightfoot CJ Jegatheesan DK Gabrys I Bennett PN. Physical activity and exercise recommendations for people receiving dialysis: a scoping review. PLoS One. (2022) 17(4):e0267290. 10.1371/journal.pone.0267290 35482797 PMC9049336

[20] Kelly C Hulme C Farragher T Clarke G. Are differences in travel time or distance to healthcare for adults in global north countries associated with an impact on health outcomes? A systematic review. BMJ Open. (2016) 6(11):e013059. 10.1136/bmjopen-2016-013059 27884848 PMC5178808

[21] Clyne N Anding-Rost K. Exercise training in chronic kidney disease-effects, expectations and adherence. Clin Kidney J. (2021) 14(Suppl 2):ii3–ii14. 10.1093/ckj/sfab012 33981415 PMC8101627

[22] Eysenbach G. What is e-health? J Med Internet Res. (2001) 3(2):E20. 10.2196/jmir.3.2.e20 11720962 PMC1761894

[23] Kelders SM Kok RN Ossebaard HC Van Gemert-Pijnen JE. Persuasive system design does matter: a systematic review of adherence to web-based interventions. J Med Internet Res. (2012) 14(6):e152. 10.2196/jmir.2104 23151820 PMC3510730

[24] Zampolini M Todeschini E Bernabeu Guitart M Hermens H Ilsbroukx S Macellari V Tele-rehabilitation: present and future. Ann Ist Super Sanita. (2008) 44(2):125–34. PMID: 18660562

[25] Kloek CJ Bossen D Veenhof C van Dongen JM Dekker J de Bakker DH. Effectiveness and cost-effectiveness of a blended exercise intervention for patients with hip and/or knee osteoarthritis: study protocol of a randomized controlled trial. BMC Musculoskelet Disord. (2014) 15:269. 10.1186/1471-2474-15-269 25103686 PMC4243525

[26] Seelman KD Hartman LM. Telerehabilitation: policy issues and research tools. Int J Telerehabil. (2009) 1(1):47–58. 10.5195/ijt.2009.6013 25945162 PMC4296776

[27] van Het Reve E Silveira P Daniel F Casati F de Bruin ED. Tablet-based strength-balance training to motivate and improve adherence to exercise in independently living older people: part 2 of a phase II preclinical exploratory trial. J Med Internet Res. (2014) 16(6):e159. 10.2196/jmir.3055 24966165 PMC4090377

[28] Silveira P van het Reve E Daniel F Casati F de Bruin ED. Motivating and assisting physical exercise in independently living older adults: a pilot study. Int J Med Inform. (2013) 82(5):325–34. 10.1016/j.ijmedinf.2012.11.015 23273420

[29] Silveira P van de Langenberg R van Het Reve E Daniel F Casati F de Bruin ED. Tablet-based strength-balance training to motivate and improve adherence to exercise in independently living older people: a phase II preclinical exploratory trial. J Med Internet Res. (2013) 15(8):e159. 10.2196/jmir.2579 23939401 PMC3742406

[30] Baez M Khaghani Far I Ibarra F Ferron M Didino D Casati F. Effects of online group exercises for older adults on physical, psychological and social wellbeing: a randomized pilot trial. PeerJ. (2017) 5:e3150. 10.7717/peerj.3150 28392983 PMC5384569

[31] Geraedts HA Zijlstra W Zhang W Spoorenberg SL Baez M Far IK A home-based exercise program driven by tablet application and mobility monitoring for frail older adults: feasibility and practical implications. Prev Chronic Dis. (2017) 14:E12. 10.5888/pcd14.160227 28152361 PMC5303650

[32] Taylor ME Close JCT Lord SR Kurrle SE Webster L Savage R Pilot feasibility study of a home-based fall prevention exercise program (StandingTall) delivered through a tablet computer (iPad) in older people with dementia. Australas J Ageing. (2020) 39(3):e278–e87. 10.1111/ajag.12717 31538401

[33] Kuijpers W Groen WG Aaronson NK van Harten WH. A systematic review of web-based interventions for patient empowerment and physical activity in chronic diseases: relevance for cancer survivors. J Med Internet Res. (2013) 15(2):e37. 10.2196/jmir.2281 23425685 PMC3636300

[34] Woodard CM Berry MJ. Enhancing adherence to prescribed exercise: structured behavioral interventions in clinical exercise programs. J Cardiopulm Rehabil. (2001) 21(4):201–9. 10.1097/00008483-200107000-00002 11508179

[35] Geraedts H Zijlstra A Bulstra SK Stevens M Zijlstra W. Effects of remote feedback in home-based physical activity interventions for older adults: a systematic review. Patient Educ Couns. (2013) 91(1):14–24. 10.1016/j.pec.2012.10.018 23194823

[36] Wantland DJ Portillo CJ Holzemer WL Slaughter R McGhee EM. The effectiveness of web-based vs. Non-web-based interventions: a meta-analysis of behavioral change outcomes. J Med Internet Res. (2004) 6(4):e40. 10.2196/jmir.6.4.e40 15631964 PMC1550624

[37] Bossen D Buskermolen M Veenhof C de Bakker D Dekker J. Adherence to a web-based physical activity intervention for patients with knee and/or hip osteoarthritis: a mixed method study. J Med Internet Res. (2013) 15(10):e223. 10.2196/jmir.2742 24132044 PMC3806355

[38] Tuot DS Boulware LE. Telehealth applications to enhance CKD knowledge and awareness among patients and providers. Adv Chronic Kidney Dis. (2017) 24(1):39–45. 10.1053/j.ackd.2016.11.017 28224941 PMC5324778

[39] Moher D Hopewell S Schulz KF Montori V Gotzsche PC Devereaux PJ CONSORT 2010 Explanation and elaboration: updated guidelines for reporting parallel group randomised trials. Br Med J. (2010) 340:c869. 10.1136/bmj.c869 20332511 PMC2844943

[40] Schulz KF Altman DG Moher D. CONSORT 2010 Statement: updated guidelines for reporting parallel group randomised trials. Br Med J. (2010) 340:c332. 10.1136/bmj.c332 20332509 PMC2844940

[41] Hoffmann TC Glasziou PP Boutron I Milne R Perera R Moher D Better reporting of interventions: template for intervention description and replication (TIDieR) checklist and guide. BMJ: Br Med J. (2014) 348:g1687. 10.1136/bmj.g1687 24609605

[42] Julious SA. Sample size of 12 per group rule of thumb for a pilot study. Pharm Stat. (2005) 4(4):287–91. 10.1002/pst.185

[43] Moore CG Carter RE Nietert PJ Stewart PW. Recommendations for planning pilot studies in clinical and translational research. Clin Transl Sci. (2011) 4(5):332–7. 10.1111/j.1752-8062.2011.00347.x 22029804 PMC3203750

[44] Baschung Pfister P Tobler-Ammann B Knols RH de Bruin ED de Bie RA. Usability and acceptance of an interactive tablet-based exercise application: a mixed methods study. Frontiers in Digital Health. (2020) 2:578281. 10.3389/fdgth.2020.578281 34713051 PMC8521963

[45] Granacher U Muehlbauer T Gschwind YJ Pfenninger B Kressig RW. [Assessment and training of strength and balance for fall prevention in the elderly: recommendations of an interdisciplinary expert panel]. Z Gerontol Geriatr. (2014) 47(6):513–26. 10.1007/s00391-013-0509-5 23912126

[46] Kenny RAM Rubenstein LZ Tinetti ME Brewer K Cameron KA Capezuti EA Prevention of Falls in Older Persons: AGS/BGS Clinical Practice Guideline. J Am Geriatr Soc. (2011) 59(1):148–57. 10.1111/j.1532-5415.2010.03234.x 21226685

[47] Panel on prevention of falls in older persons AGS, British geriatrics S. Summary of the updated American geriatrics society/British geriatrics society clinical practice guideline for prevention of falls in older persons. J Am Geriatr Soc. (2011) 59(1):148–57. 10.1111/j.1532-5415.2010.03234.x 21226685

[48] Sherrington C Whitney JC Lord SR Herbert RD Cumming RG Close JC. Effective exercise for the prevention of falls: a systematic review and meta-analysis. J Am Geriatr Soc. (2008) 56(12):2234–43. 10.1111/j.1532-5415.2008.02014.x 19093923

[49] Koh KP Fassett RG Sharman JE Coombes JS Williams AD. Effect of intradialytic versus home-based aerobic exercise training on physical function and vascular parameters in hemodialysis patients: a randomized pilot study. Am J Kidney Dis. (2010) 55(1):88–99. 10.1053/j.ajkd.2009.09.025 19932545

[50] Kouidi E Iacovides A Iordanidis P Vassiliou S Deligiannis A Ierodiakonou C Exercise renal rehabilitation program: psychosocial effects. Nephron. (1997) 77(2):152–8. 10.1159/000190266 9346380

[51] Malagoni AM Catizone L Mandini S Soffritti S Manfredini R Boari B Acute and long-term effects of an exercise program for dialysis patients prescribed in hospital and performed at home. J Nephrol. (2008) 21(6):871–8. PMID: 19034871

[52] Ortega-Perez de Villar L Martinez-Olmos FJ Perez-Dominguez FB Benavent-Caballer V Montanez-Aguilera FJ Mercer T Comparison of intradialytic versus home-based exercise programs on physical functioning, physical activity level, adherence, and health-related quality of life: pilot study. Sci Rep. (2020) 10(1):8302. 10.1038/s41598-020-64372-y 32427935 PMC7237690

[53] Mañas A Gómez-Redondo P Valenzuela PL Morales JS Lucía A Ara I. Unsupervised home-based resistance training for community-dwelling older adults: a systematic review and meta-analysis of randomized controlled trials. Ageing Res Rev. (2021) 69:101368. 10.1016/j.arr.2021.101368 34022464

[54] Davis FD. Perceived usefulness, perceived ease of use, and user acceptance of InformationTechnology. MIS Qaurterly. (1989) 13(3):319–40. 10.2307/249008

[55] Davis FD Bagozzi RP Warshaw PR. User acceptance of computer technology: a comparison of two theoretical models. Manage Sci. (1989) 35(8):982–1003. 10.1287/mnsc.35.8.982

[56] Panday R Rachmat B. Technology readiness acceptance model analysis on project management operations. Int j bus Manag. (2019) 4(3):117–32.

[57] Caplin B Kumar S Davenport A. Patients’ perspective of haemodialysis-associated symptoms. Nephrol Dial Transplant. (2011) 26(8):2656–63. 10.1093/ndt/gfq763 21212166

[58] Maggio M Ceda GP Ticinesi A De Vita F Gelmini G Costantino C Instrumental and non-instrumental evaluation of 4-meter walking speed in older individuals. PLoS One. (2016) 11(4):e0153583. 10.1371/journal.pone.0153583 27077744 PMC4831727

[59] Cruz-Jentoft AJ Bahat G Bauer J Boirie Y Bruyere O Cederholm T Sarcopenia: revised European consensus on definition and diagnosis. Age Ageing. (2019) 48(1):16–31. 10.1093/ageing/afy169 PMC6322506 30312372

[60] Innes E. Handgrip strength testing: a review of the literature. Aust Occup Ther J. (1999) 46:120–40. 10.1046/j.1440-1630.1999.00182.x

[61] Guralnik JM Simonsick EM Ferrucci L Glynn RJ Berkman LF Blazer DG A short physical performance battery assessing lower extremity function: association with self-reported disability and prediction of mortality and nursing home admission. J Gerontol. (1994) 49(2):M85–94. 10.1093/geronj/49.2.M85 8126356

[62] Pavasini R Guralnik J Brown JC di Bari M Cesari M Landi F Short physical performance battery and all-cause mortality: systematic review and meta-analysis. BMC Med. (2016) 14(1):215. 10.1186/s12916-016-0763-7 28003033 PMC5178082

[63] Ozalevli S Ozden A Itil O Akkoclu A. Comparison of the sit-to-stand test with 6 min walk test in patients with chronic obstructive pulmonary disease. Respir Med. (2007) 101(2):286–93. 10.1016/j.rmed.2006.05.007 16806873

[64] Strassmann A Steurer-Stey C Lana KD Zoller M Turk AJ Suter P Population-based reference values for the 1-min sit-to-stand test. Int J Public Health. (2013) 58(6):949–53. 10.1007/s00038-013-0504-z 23974352

[65] Podsiadlo D Richardson S. The timed “up & go": a test of basic functional mobility for frail elderly persons. J Am Geriatr Soc. (1991) 39(2):142–8. 10.1111/j.1532-5415.1991.tb01616.x 1991946

[66] Barry E Galvin R Keogh C Horgan F Fahey T. Is the timed up and go test a useful predictor of risk of falls in community dwelling older adults: a systematic review and meta-analysis. BMC Geriatr. (2014) 14:14. 10.1186/1471-2318-14-14 24484314 PMC3924230

[67] Smith E Walsh L Doyle J Greene B Blake C. Effect of a dual task on quantitative timed up and go performance in community-dwelling older adults: a preliminary study. Geriatr Gerontol Int. (2017) 17(8):1176–82. 10.1111/ggi.12845 27427514

[68] Lacson E Jr., Xu J Lin SF Dean SG Lazarus JM Hakim RM. A comparison of SF-36 and SF-12 composite scores and subsequent hospitalization and mortality risks in long-term dialysis patients. Clin J Am Soc Nephrol. (2010) 5(2):252–60. 10.2215/CJN.07231009 20019120 PMC2827595

[69] Ware J Kosinski M Jr Keller SD. A 12-item short-form health survey: construction of scales and preliminary tests of reliability and validity. Med Care. (1996) 34(3):220–33. 10.1097/00005650-199603000-00003 8628042

[70] Ware JE Jr., Sherbourne CD. The MOS 36-item short-form health survey (SF-36). I. Conceptual framework and item selection. Med Care. (1992) 30(6):473–83. 10.1097/00005650-199206000-00002 1593914

[71] Kodraliu G Mosconi P Groth N Carmosino G Perilli A Gianicolo EA Subjective health status assessment: evaluation of the Italian version of the SF-12 health survey. Results from the MiOS project. J Epidemiol Biostat. (2001) 6(3):305–16. 10.1080/135952201317080715 11437095

[72] de Morton NA Davidson M Keating JL. The de morton mobility Index (DEMMI): an essential health index for an ageing world. Health Qual Life Outcomes. (2008) 6:63. 10.1186/1477-7525-6-63 18713451 PMC2551589

[73] Sim J. Should treatment effects be estimated in pilot and feasibility studies? Pilot Feasibility Stud. (2019) 5:107. 10.1186/s40814-019-0493-7 31485336 PMC6712606

[74] Teresi JA Yu X Stewart AL Hays RD. Guidelines for designing and evaluating feasibility pilot studies. Med Care. (2022) 60(1):95–103. 10.1097/MLR.0000000000001664 34812790 PMC8849521

[75] Cohen J. Statistical power analysis for the behavioural sciences. 2nd ed, edn. New York: Lawrence Erlbaum Associates (1988).

[76] Hudon C Fortin M Soubhi H. Abbreviated guidelines for scoring the cumulative illness rating scale (CIRS) in family practice. J Clin Epidemiol. (2007) 60(2):212. 10.1016/j.jclinepi.2005.12.021 17208130

[77] Kirkhus L Jordhoy M Saltyte Benth J Rostoft S Selbaek G Jensen Hjermstad M Comparing comorbidity scales: attending physician score versus the cumulative illness rating scale for geriatrics. J Geriatr Oncol. (2016) 7(2):90–8. 10.1016/j.jgo.2015.12.003 26739557

[78] Miller MD Paradis CF Houck PR Mazumdar S Stack JA Rifai AH Rating chronic medical illness burden in geropsychiatric practice and research: application of the cumulative illness rating scale. Psychiatry Res. (1992) 41(3):237–48. 10.1016/0165-1781(92)90005-N 1594710

[79] Baggetta R D'Arrigo G Torino C ElHafeez SA Manfredini F Mallamaci F Effect of a home based, low intensity, physical exercise program in older adults dialysis patients: a secondary analysis of the EXCITE trial. BMC Geriatr. (2018) 18(1):248. 10.1186/s12877-018-0938-5 30342464 PMC6196029

[80] Tao X Chow SKY Wong FKY. A nurse-led case management program on home exercise training for hemodialysis patients: a randomized controlled trial. Int J Nurs Stud. (2015) 52(6):1029–41. 10.1016/j.ijnurstu.2015.03.013 25840898

[81] Bennett PN Peter J Wang W Street M. Attitudes of nephrology nurses toward patient exercise during hemodialysis. Nephrol Nurs J. (2016) 43(4):331–7. PMID: 30550060

[82] Fournier J. Nurse-led home exercise programme improves physical function for people receiving haemodialysis. Evid Based Nurs. (2016) 19(1):12. 10.1136/eb-2015-102156 26376906

[83] Ufficio federale di statistica. Indagine sull'uso di internet. 2021.

[84] Baschung Pfister P Knols RH de Bie RA de Bruin ED. Feasibility of a blended therapy approach in the treatment of patients with inflammatory myopathies. Archives of Physiotherapy. (2021) 11(1):14. 10.1186/s40945-021-00108-z 34039438 PMC8157458

[85] Bjerke MB Renger R. Being smart about writing SMART objectives. Eval Program Plann. (2017) 61:125–7. 10.1016/j.evalprogplan.2016.12.009 28056403

[86] Evangelidis N Craig J Bauman A Manera K Saglimbene V Tong A. Lifestyle behaviour change for preventing the progression of chronic kidney disease: a systematic review. BMJ Open. (2019) 9(10):e031625. 10.1136/bmjopen-2019-031625 31662393 PMC6830616

[87] Hawley-Hague H Lasrado R Martinez E Stanmore E Tyson S. A scoping review of the feasibility, acceptability, and effects of physiotherapy delivered remotely. Disabil Rehabil. (2022) 1–17. 10.1080/09638288.2022.2138574 36325612

[88] O'Cathain A Croot L Duncan E Rousseau N Sworn K Turner KM Guidance on how to develop complex interventions to improve health and healthcare. BMJ Open. (2019) 9(8):e029954. 10.1136/bmjopen-2019-029954 PMC6701588 31420394

[89] Liu-Ambrose T Davis JC Best JR Dian L Madden K Cook W Effect of a home-based exercise program on subsequent falls among community-dwelling high-risk older adults after a fall: a randomized clinical trial. JAMA. (2019) 321(21):2092–100. 10.1001/jama.2019.5795 31162569 PMC6549299

[90] Kamide N Shiba Y Shibata H. Effects on balance, falls, and bone mineral density of a home-based exercise program without home visits in community-dwelling elderly women: a randomized controlled trial. J Physiol Anthropol. (2009) 28(3):115–22. 10.2114/jpa2.28.115 19483372

[91] Declaration of Helsinki (2013). [Available at: https://www.wma.net/policies-post/wma-declaration-of-helsinki-ethical-principles-for-medical-research-involving-human-subjects/

