# Erratum: A blended e-health intervention for improving functional capacity in elderly patients on haemodialysis: A feasibility study

**DOI:** 10.3389/fdgth.2023.1171888

**Published:** 2023-03-16

**Authors:** Frontiers Production Office

**Affiliations:** Frontiers Media SA, Lausanne, Switzerland

**Keywords:** tablet-computer, exercise, haemodialysis, elderly, e-health, blended intervention, physiotherapy

An Erratum on A blended e-health intervention for improving functional capacity in elderly patients on haemodialysis: A feasibility study By Zemp DD, Baschung Pfister P, Knols R, Quadri P, Bianchi G, Giunzioni D, Lavorato S, Giannini O and de Bruin E. (2022) Front. Digit. Health. 4:1054932. doi: 10.3389/fdgth.2022.1054932

An omission to the **Funding** section of the original article was made in error. The following sentence has been added: “Open access funding was provided by ETH Zurich”.

The original article has been updated.

