# Supplementary material for: A blended e-health intervention for improving functional capacity in elderly patients on haemodialysis: A feasibility study
Source: Front Digit Health. 2022 Dec 6;4:1054932. doi: 10.3389/fdgth.2022.1054932 (PMC9763896; doi:10.3389/fdgth.2022.1054932)
Supplement: Supplementary file 1 [file Datasheet1.docx]

Supplementary Material

# Supplementary Table S1 CONSORT Checklist


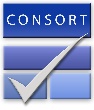


CONSORT 2010 checklist of information to include when reporting a pilot or feasibility trial*

| Section/Topic | Item No | Checklist item | Reported on page No |
| --- | --- | --- | --- |
| Title and abstract |  |  |  |
|  | 1a | Identification as a pilot or feasibility randomised trial in the title | 1 |
|  | 1b | Structured summary of pilot trial design, methods, results, and conclusions (for specific guidance see CONSORT abstract extension for pilot trials) | 1-2 |
| Introduction |  |  |  |
| Background and objectives | 2a | Scientific background and explanation of rationale for future definitive trial, and reasons for randomised pilot trial | 2-3 |
|  | 2b | Specific objectives or research questions for pilot trial | 3 |
| Methods |  |  |  |
| Trial design | 3a | Description of pilot trial design (such as parallel, factorial) including allocation ratio | 3 |
|  | 3b | Important changes to methods after pilot trial commencement (such as eligibility criteria), with reasons | N/A |
| Participants | 4a | Eligibility criteria for participants | 3 |
|  | 4b | Settings and locations where the data were collected | 3 |
|  | 4c | How participants were identified and consented | 3 |
| Interventions | 5 | The interventions for each group with sufficient details to allow replication, including how and when they were actually administered | 3-4 |
| Outcomes | 6a | Completely defined prespecified assessments or measurements to address each pilot trial objective specified in 2b, including how and when they were assessed | 3-5 |
|  | 6b | Any changes to pilot trial assessments or measurements after the pilot trial commenced, with reasons | N/A |
|  | 6c | If applicable, prespecified criteria used to judge whether, or how, to proceed with future definitive trial | N/A |
| Sample size | 7a | Rationale for numbers in the pilot trial | 3 |
|  | 7b | When applicable, explanation of any interim analyses and stopping guidelines | N/A |
| *Randomisation:* |  |  |  |
| Sequence  generation | 8a | Method used to generate the random allocation sequence | N/A |
|  | 8b | Type of randomisation(s); details of any restriction (such as blocking and block size) | N/A |
| Allocation concealment mechanism | 9 | Mechanism used to implement the random allocation sequence (such as sequentially numbered containers), describing any steps taken to conceal the sequence until interventions were assigned | N/A |
| Implementation | 10 | Who generated the random allocation sequence, who enrolled participants, and who assigned participants to interventions | N/A |
| Blinding | 11a | If done, who was blinded after assignment to interventions (for example, participants, care providers, those assessing outcomes) and how | N/A |
|  | 11b | If relevant, description of the similarity of interventions | N/A |
| Statistical methods | 12 | Methods used to address each pilot trial objective whether qualitative or quantitative | 5 |
| Results | | | |
| Participant flow (a diagram is strongly recommended) | 13a | For each group, the numbers of participants who were approached and/or assessed for eligibility, randomly assigned, received intended treatment, and were assessed for each objective | 6-7 |
|  | 13b | For each group, losses and exclusions after randomisation, together with reasons | 6-7 |
| Recruitment | 14a | Dates defining the periods of recruitment and follow-up | 5 |
|  | 14b | Why the pilot trial ended or was stopped | - |
| Baseline data | 15 | A table showing baseline demographic and clinical characteristics for each group | 5-6 |
| Numbers analysed | 16 | For each objective, number of participants (denominator) included in each analysis. If relevant, these numbers should be by randomised group | 5-8 |
| Outcomes and estimation | 17 | For each objective, results including expressions of uncertainty (such as 95% confidence interval) for any  estimates. If relevant, these results should be by randomised group | 5-8 |
| Ancillary analyses | 18 | Results of any other analyses performed that could be used to inform the future definitive trial | N/A |
| Harms | 19a | All important harms or unintended effects in each group (for specific guidance see CONSORT for harms) | N/A |
|  | 19b | If relevant, other important unintended consequences | N/A |
| Discussion | | | |
| Limitations | 20 | Pilot trial limitations, addressing sources of potential bias and remaining uncertainty about feasibility | 10 |
| Generalisability | 21 | Generalisability (applicability) of pilot trial methods and findings to future definitive trial and other studies | 8-9 |
| Interpretation | 22a | Interpretation consistent with pilot trial objectives and findings, balancing potential benefits and harms, and considering other relevant evidence | 8-9 |
|  | 22b | Implications for progression from pilot to future definitive trial, including any proposed amendments | 8-9 |
| **Other information** | | | |
| Registration | 23 | Registration number for pilot trial and name of trial registry | 10 |
| Protocol | 24 | Where the pilot trial protocol can be accessed, if available | N/A |
| Funding | 25 | Sources of funding and other support (such as supply of drugs), role of funders | 10-11 |
| Ethics | 26 | Ethical approval or approval by research review committee, confirmed with reference number | 10 |

Citation: Eldridge SM, Chan CL, Campbell MJ, Bond CM, Hopewell S, Thabane L, et al. CONSORT 2010 statement: extension to randomised pilot and feasibility trials. BMJ. 2016;355.

*We strongly recommend reading this statement in conjunction with the CONSORT 2010, extension to randomised pilot and feasibility trials, Explanation and Elaboration for important clarifications on all the items. If relevant, we also recommend reading CONSORT extensions for cluster randomised trials, non-inferiority and equivalence trials, non-pharmacological treatments, herbal interventions, and pragmatic trials. Additional extensions are forthcoming: for those and for up to date references relevant to this checklist, see [www.consort-statement.org](http://www.consort-statement.org).

# Supplementary Table S2 TIDieR checklist.

**
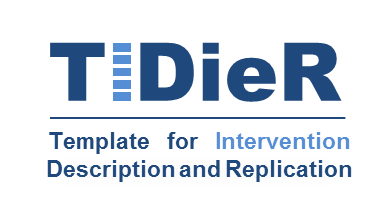
The TIDieR (Template for Intervention Description and Replication) Checklist*:**

Information to include when describing an intervention and the location of the information

| **Item number** | **Item** | **Where located **** | |
| --- | --- | --- | --- |
|  |  | Primary paper (page or appendix  number) | Other ^†^ (details) |
|  | **BRIEF NAME** |  |  |
| **1.** | Provide the name or a phrase that describes the intervention. | 3 |  |
|  | **WHY** |  |  |
| **2.** | Describe any rationale, theory, or goal of the elements essential to the intervention. | 2-3 |  |
|  | **WHAT** |  |  |
| **3.** | Materials: Describe any physical or informational materials used in the intervention, including those provided to participants or used in intervention delivery or in training of intervention providers. Provide information on where the materials can be accessed (e.g. online appendix, URL). | 3-4 |  |
| **4.** | Procedures: Describe each of the procedures, activities, and/or processes used in the intervention, including any enabling or support activities. | 3-4 |  |
|  | **WHO PROVIDED** |  |  |
| **5.** | For each category of intervention provider (e.g. psychologist, nursing assistant), describe their expertise, background and any specific training given. | 4 |  |
|  | **HOW** |  |  |
| **6.** | Describe the modes of delivery (e.g. face-to-face or by some other mechanism, such as internet or telephone) of the intervention and whether it was provided individually or in a group. | 3-4 |  |
|  | **WHERE** |  |  |
| **7.** | Describe the type(s) of location(s) where the intervention occurred, including any necessary infrastructure or relevant features. | 3-4 |  |
|  | **WHEN and HOW MUCH** |  |  |
| **8.** | Describe the number of times the intervention was delivered and over what period of time including the number of sessions, their schedule, and their duration, intensity or dose. | 3-4 |  |
|  | **TAILORING** |  |  |
| **9.** | If the intervention was planned to be personalised, titrated or adapted, then describe what, why, when, and how. | 3-4 |  |
|  | **MODIFICATIONS** |  |  |
| **10.^ǂ^** | If the intervention was modified during the course of the study, describe the changes (what, why, when, and how). | N/A |  |
|  | **HOW WELL** |  |  |
| **11.** | Planned: If intervention adherence or fidelity was assessed, describe how and by whom, and if any strategies were used to maintain or improve fidelity, describe them. | 4 |  |
| 12.ǂ | Actual: If intervention adherence or fidelity was assessed, describe the extent to which the intervention was delivered as planned. | 6-7 |  |

^**^ Authors - use N/A if an item is not applicable for the intervention being described. Reviewers – use ‘?’ if information about the element is not reported/not sufficiently reported.

^†^ If the information is not provided in the primary paper, give details of where this information is available. This may include locations such as a published protocol or other published papers (provide citation details) or a website (provide the URL).

^ǂ^ If completing the TIDieR checklist for a protocol, these items are not relevant to the protocol and cannot be described until the study is complete.

^*^ We strongly recommend using this checklist in conjunction with the TIDieR guide (see BMJ 2014;348:g1687) which contains an explanation and elaboration for each item.

^*^ The focus of TIDieR is on reporting details of the intervention elements (and where relevant, comparison elements) of a study. Other elements and methodological features of studies are covered by other reporting statements and checklists and have not been duplicated as part of the TIDieR checklist. When a randomised trial is being reported, the TIDieR checklist should be used in conjunction with the CONSORT statement (see [www.consort-statement.org](http://www.consort-statement.org)) as an extension of Item 5 of the CONSORT 2010 Statement. When a clinical trial protocol is being reported, the TIDieR checklist should be used in conjunction with the SPIRIT statement as an extension of Item 11 of the SPIRIT 2013 Statement (see [www.spirit-statement.org](http://www.spirit-statement.org)). For alternate study designs, TIDieR can be used in conjunction with the appropriate checklist for that study design (see [www.equator-network.org](http://www.equator-network.org)).

# Supplementary File S3 Assessments battery (references in the main text).

4m walk Test

The goal of the test is to measure comfortable pace gait speed from a standing start. The patient is asked to begin walking from the start line and stop at least 2m after the end of the 4m line. The PT measures with a stopwatch the time needed to walk the 4m from a standing start.

Timed Get up and Go Test under single and dual-task

The test measures the time required to stand up, walk 3m, turn, walk back, and sit down. In the TUG under motor dual-task, subjects have to carry a glass of water. In the cognitive dual-task condition the subjects have to count down by threes from 100.

Short Physical Performance Battery

The SPPB includes tests of walking speed, standing balance and chair stand. Each SPPB component test (gait, balance and chair stand) is scored from 0 to 4, with a score of 0 representing inability to carry out the test, and 4 as the best performance. For balance, the participants are asked to maintain their feet side-by-side, in semi-tandem and tandem positions for 10s each. For gait, a 4m walk at the participants’ usual speed was timed from a standing start. For the chair stand test, participants were asked to stand up and sit down five times as quickly as possible.

60-second chair stand

The 60-second chair stand measures the number of times that the patient rises to a full stand from a seated position with arms folded within 60 seconds at a comfortable pace according to the patient's own rhythm. It is important that the same or a similar chair is used on each occasion, as the score may be influenced by the height of the chair.

The Short Form health survey

The Short Form health survey, SF-12, is a commonly used instrument to measure the health-related quality of life at various ages. The instrument is a shorter version of the SF-36, developed by Ware and Sherbourne. The SF-12 uses only 12 questions to measure functional health and wellbeing from the patient’s perspective. The original objective was to develop a short, generic health-status measure that reproduces the 2 summary scores of the SF-36 (the physical component summary (PCS) score and the mental component summary (MCS) score). The SF-12 is validated for long-term dialysis patients.

The de Morton Mobility Index

The de Morton Mobility Index (DEMMI) is a unidimensional measure of mobility. The DEMMI consists of 15 items. Eleven items are dichotomous (scored 0 or 1) and four are scored 0, 1 or 2. The DEMMI is administered by direct observation by a clinician of the performance of the patient on 15 hierarchical mobility activities (three are bed-based, three chair-based, four involve static balance, two are walking-related and three involve dynamic balance). Patients are scored on their ability as either able/unable or able/partially able/unable to perform the tasks.

# Supplementary Table S4 Effect of the intervention on functional capacities and health related parameters.

|  |  |
| --- | --- |
|  |  |
|  |  |
|  |  |
|  |  |
